# Supplementary material for: Structural basis underlying the synergism of NADase and SLO during group A Streptococcus infection
Source: Commun Biol. 2023 Jan 31;6:124. doi: 10.1038/s42003-023-04502-0 (PMC9887584; doi:10.1038/s42003-023-04502-0)
Supplement: Supplementary file 2 — Supplementary Information [file 42003_2023_4502_MOESM2_ESM.pdf]

Supplementary Information for

Structural basis underlying the synergism of NADase and SLO during Group A *Streptococcus* infection

Wei-Jiun Tsai, Yi-Hsin Lai, Yong-An Shi, Michal Hammel, Anthony P. Duff, Andrew E. Whitten, Karyn L. Wilde, Chun-Ming Wu, Robert Knott, U-Ser Jeng, Chia-Yu Kang, Chih-Yu Hsu, Jian-Li Wu, Pei-Jane Tsai, Chuan Chiang-Ni, Jiunn-Jong Wu, Yee-Shin Lin, Ching-Chuan Liu, Toshiya Senda, Shuying Wang

Shuying Wang
Email: sswang23@mail.ncku.edu.tw

This PDF file includes:

Supplementary Tables 1 to 2
Supplementary Figures 1 to 12
Supplementary References

Supplementary Table 1. SAXS data collection and parameters.

Data Collection	NADase	NADase/SLO complex
Beamline	SIBYLS beamline 12.3.1	
Beam energy	11kEV	
Wavelength (Å)	1.03	
Sample-detector distance (m)	1.5	
Detector	Pilatus 2M	
Exposure time (s)	3	
q range ($Å^{-1}$)	0.01 - 0.4	0.01 - 0.4
Temperature (K)	293	
Data Analysis		
R_g (real) (Å)	29.8 ± 0.1	48.5 ± 0.1
R_g (reciprocal) (Å)	29.7 ± 0.1	47.2 ± 1.4
D_{max} (Å)	~103	~184
Porod Volume ($Å^3$)	75676	174948
MW estimation (kDa)[a]	~47.8	~108.8
MW prediction (kDa)[b]	~47.2	~110.0
Porod Exponent	3.8	3.5
Software		
Primary data reduction	SCÅTTER	
Data processing	SCÅTTER	
Rigid body modelling	BILBOMD	
3D graphics representations	Chimera	
SASBDB ID	SASDM47	SASDM57

[a]Molecular weight estimated using web server SAXSMoW.
[b]Molecular weight predicted from the protein sequences.

Supplementary Table 2. SANS data collection on NADase/SLO complex.

Data Collection Parameters	SANS
Instrument	QUOKKA (ANSTO)
Beam geometry	Point
Wavelength (Å)	5.00
Sample-detector distance (m)	2.047 (short)<br>12.046 (long)
q range ($Å^{-1}$) Exposure time (s)	0.03 - 0.50 (short)<br>0.005 - 0.06 (long)
Exposure time (s)	7200 (short)<br>14400 (long)
Measurement type	Neutron contrast variation, Hellma 120-QS 1.0 mm quartz cells
Temperature (K)	283
Absolute intensity calibration	incident beam intensity
Sample details	
Extinction coefficient (A_{280}, 0.1% w/v)	1.044
Partial specific volume (cm^3g^{-1})	0.730
Contrast, $\Delta\rho$ (10^{10} cm^2)	4.17 (0%); 3.07 (20%); 1.86 (42%); 0.87 (80%); 1.32 (100%)
Protein concentration (mg/ml)*	3.5 (0%); 3.5 (20%);<br>3.5 (42%); 3.5 (80%);<br>3.5 (100%)
Structural parameters	
I(0) (cm^{-1}) [from p(r)]	0.392 ± 0.007 (0%)<br>0.186 ± 0.006 (20%)<br>0.060 ± 0.003 (42%)<br>0.030 ± 0.003 (60%)<br>0.207 ± 0.002 (100%)
R_g (Å) [from p(r)]	48.8 ± 2.4 (0%)<br>46.0 ± 5.0 (20%)<br>29.0 ± 5.0 (42%)<br>17.0 ± 14.0 (60%)<br>45.5 ± 1.0 (100%)
D_{max} (Å)	190 ± 10

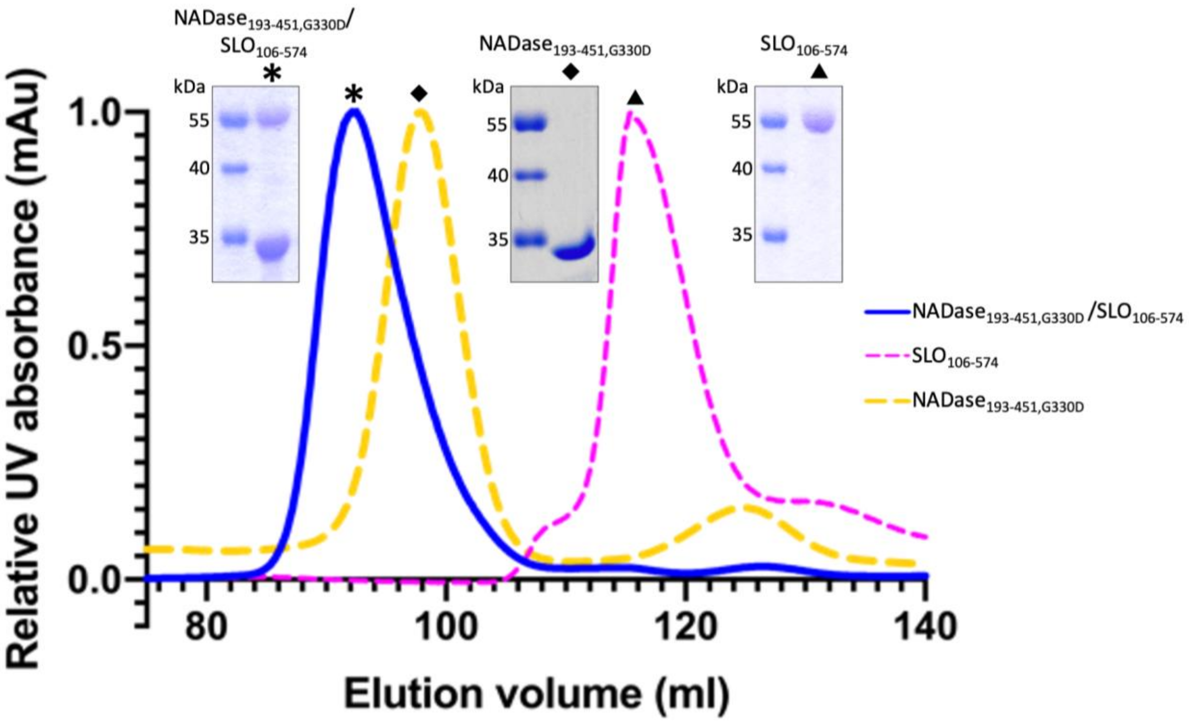

Supplementary Figure 1. Complex formation of $NADase_{193-451,G330D}$ and $SLO_{106-574}$. Size exclusion chromatographic profile and SDS-PAGE showed $NADase_{193-451,G330D}$ and $SLO_{106-574}$ were co-eluted from a single peak and formed the $NADase_{193-451,G330D}/SLO_{106-574}$ complex. The elution peak of $SLO_{106-574}$ was delayed, consistent with the behavior of SLO[1].

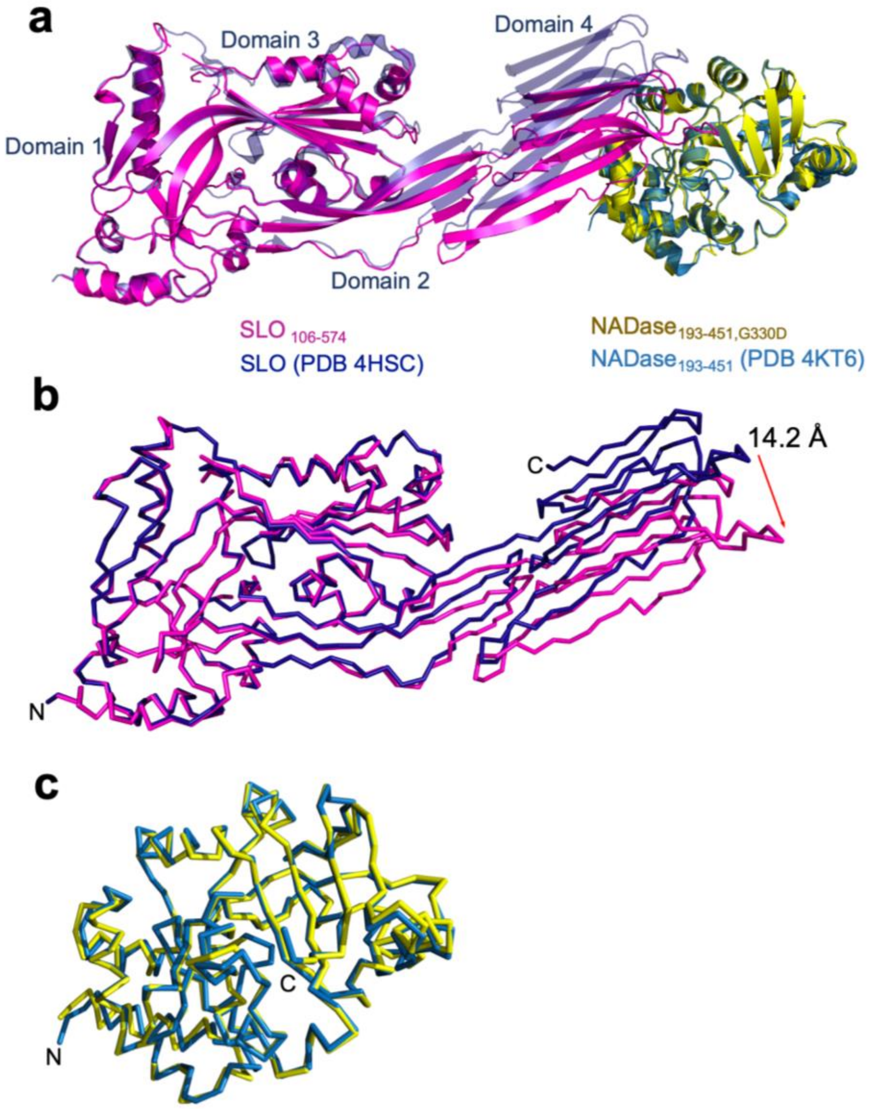

Supplementary Figure 2. Structural comparison of NADase$_{193\text{-}451}$ and SLO$_{106\text{-}574}$ to the NADase$_{193\text{-}451,G330D}$/SLO$_{106\text{-}574}$ complex. **a** Crystal structure of NADase$_{193\text{-}451,G330D}$/SLO$_{106\text{-}574}$ superposed with the structure of NADase$_{193\text{-}451}$ in complex with IFS (PDB 4KT6, transparent sky blue) and the structure of SLO-alone (PDB 4HSC, transparent dark blue). **b** Superimposition of C_α atoms of SLO-alone structure (dark blue) with SLO in complex with NADase$_{193\text{-}451,G330D}$ (magenta). **c** Superimposition of C_α atoms of the structure of SLO-bound NADase$_{193\text{-}451,G330D}$ (yellow) with the structure of NADase$_{193\text{-}451}$ in complex with IFS (sky blue).

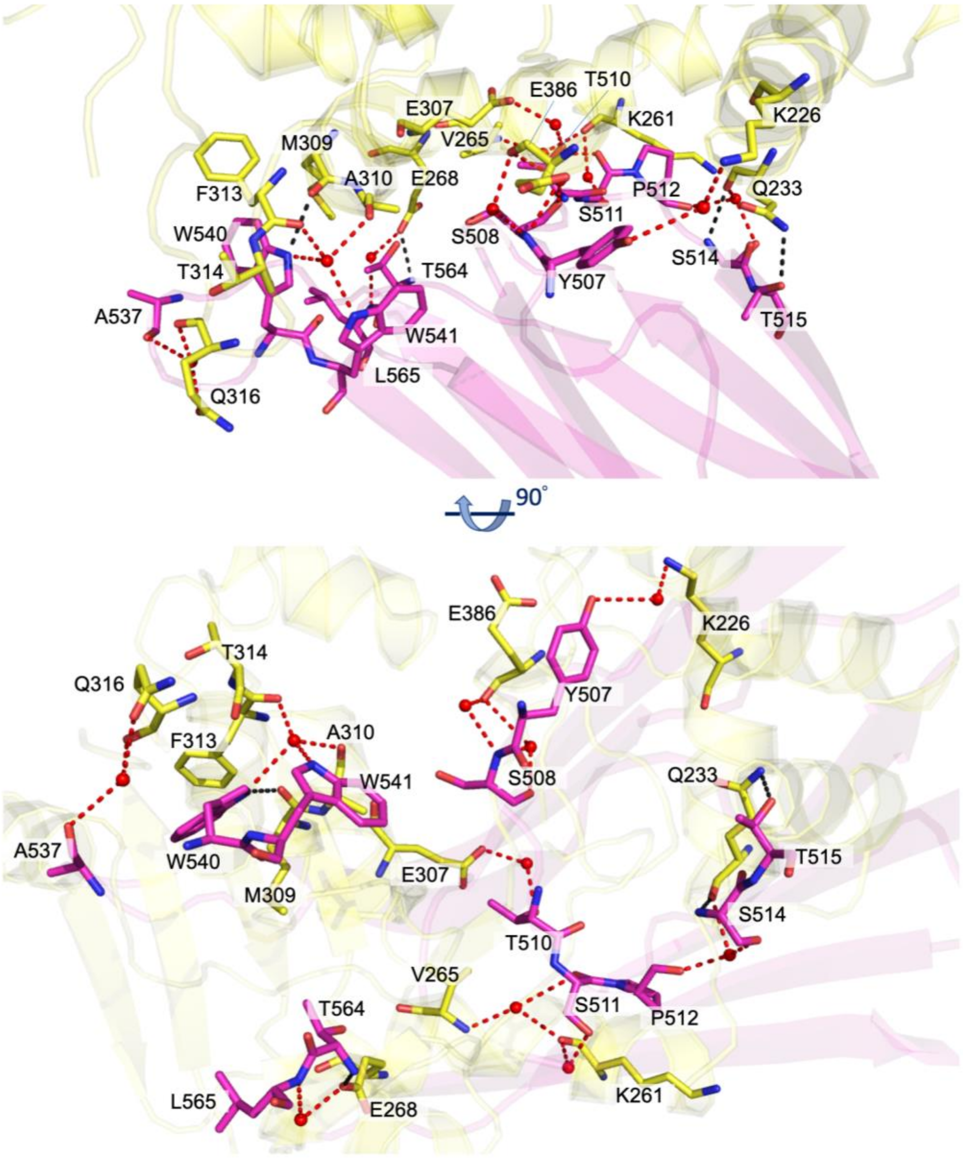

Supplementary Figure 3. The hydrogen bonding network at the NADase$_{193-451,G330D}$/SLO$_{106-574}$ Interface. The interacting residues of NADase (yellow) and SLO (magenta) are shown as sticks. NADase residues Q233, E268, and M309 are hydrogen-bonded with SLO residues S514, T515, T564, and W540, respectively (black dash). Ten water molecules (red sphere) bridge NADase and SLO with 26 pairs of hydrogen bonds (red dash).

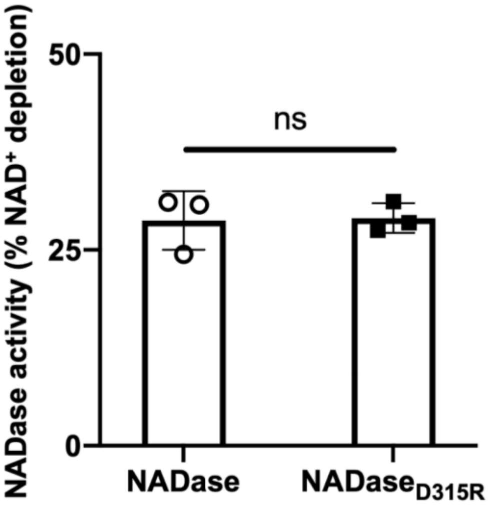

Supplementary Figure 4. Enzymatic activities of NADase and NADase$_{D315R}$. Data from three independent experiments are presented as means ± standard deviation.

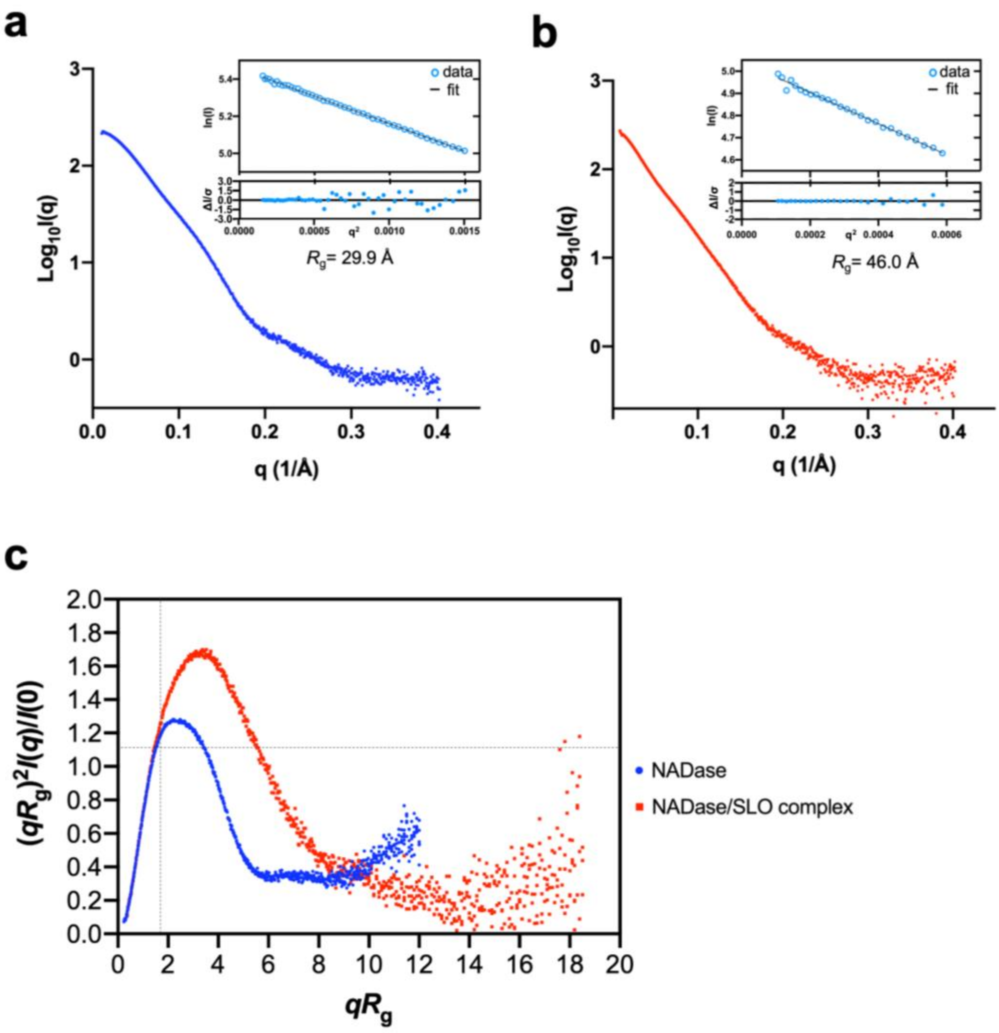

Supplementary Figure 5. SAXS data. Experimental scattering profiles of NADase (**a**) and NADase/SLO complex (**b**). *Insets* on scattering curves show the Guinier plot with the linear fit and the corresponding radius of gyration (R_g) values. **c** Dimensionless Kratky plots of NADase and the NADase/SLO complex. Dotted lines are drawn at $qR_g=\sqrt{3}$ and $(qR_g)^2I(q)/I(0) = 1.104$.

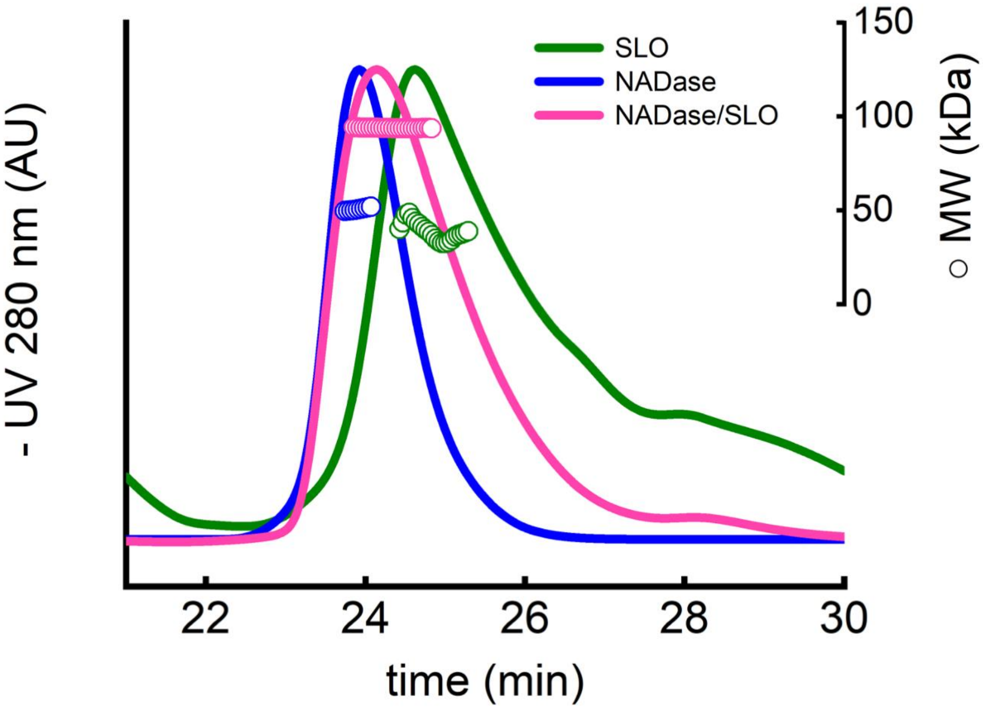

Supplementary Figure 6. SEC-MALS analysis. SEC-MALS chromatograms for SLO (dark green), NADase (blue) and NADase/SLO (pink). Solid lines represent the UV signal in arbitrary units (AU). Symbols represent molecular mass determined by MALS versus elution time.

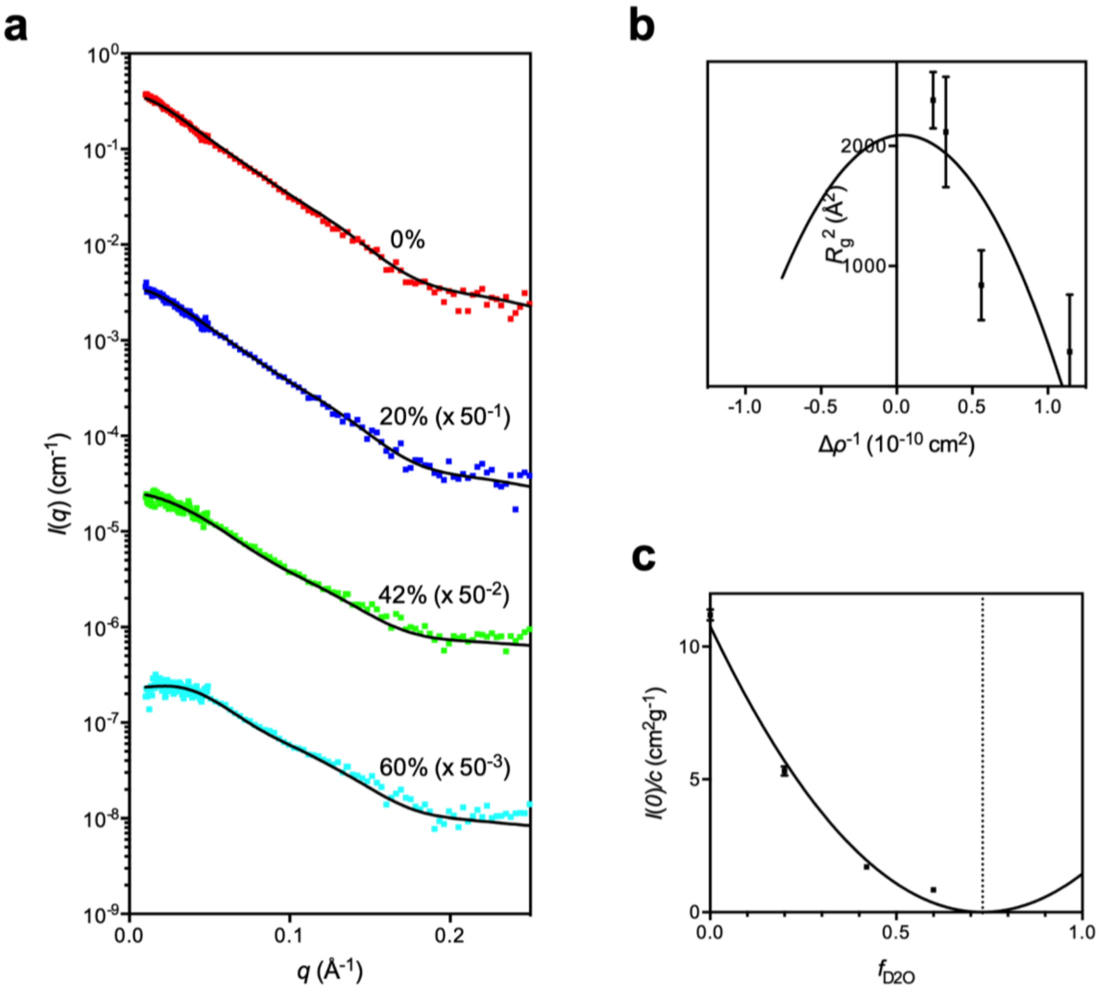

Supplementary Figure 7. SANS results. a SANS data (offset for clarity) collected from the ^{D}NADase/SLO complex with the multistate model scattering curves overlaid (solid black line): 0% (χ^2 = 0.39, red, on absolute scale); 20% (χ^2 = 0.19, blue, offset by a factor of 50^{-1}); 42% (χ^2 = 0.19, green, offset by a factor of 50^{-2}); 60% (χ^2 = 0.19, cyan, offset by a factor of 50^{-3}). The multistate model used to fit the neutron scattering data is identical to that used to fit the SAXS data (9% compact state, 53% extended state, 38% of free NADase). The I(0) value for the 100% D_2O sample was anomalously high, indicating higher order association, and was excluded from further analysis. The 0% D_2O sample was analysed by SAXS and verified to be consistent with the data shown in Supplementary Figure 5. **b** Stuhrmann plot for the NADase/SLO complex, conforming to equation $R_g^2 = R_m^2 + \alpha\Delta\rho^{-1} - \beta\Delta\rho^{-2}$, where R_m is the radius of gyration

of that object with homogenous contrast, and α and β are related to contrast fluctuations within the object. The values obtained from a fit to the plot (solid black line) are: R_m = 46 ± 6; α = 100 ± 1500; and β = 1900 ± 1300. The precision of the parameters is low as there is no radius of gyration value measured where the particle contrast is negative to restrain the shape of the parabola. **c** A plot I(0) normalized by concentration as a function of D_2O content of the supporting solvent. The plot is parabolic in shape and reveals that the match-point of the entire complex is 73% D_2O (vertical dotted line). This is close to the value calculated based on the sequence and deuteration level (76%).

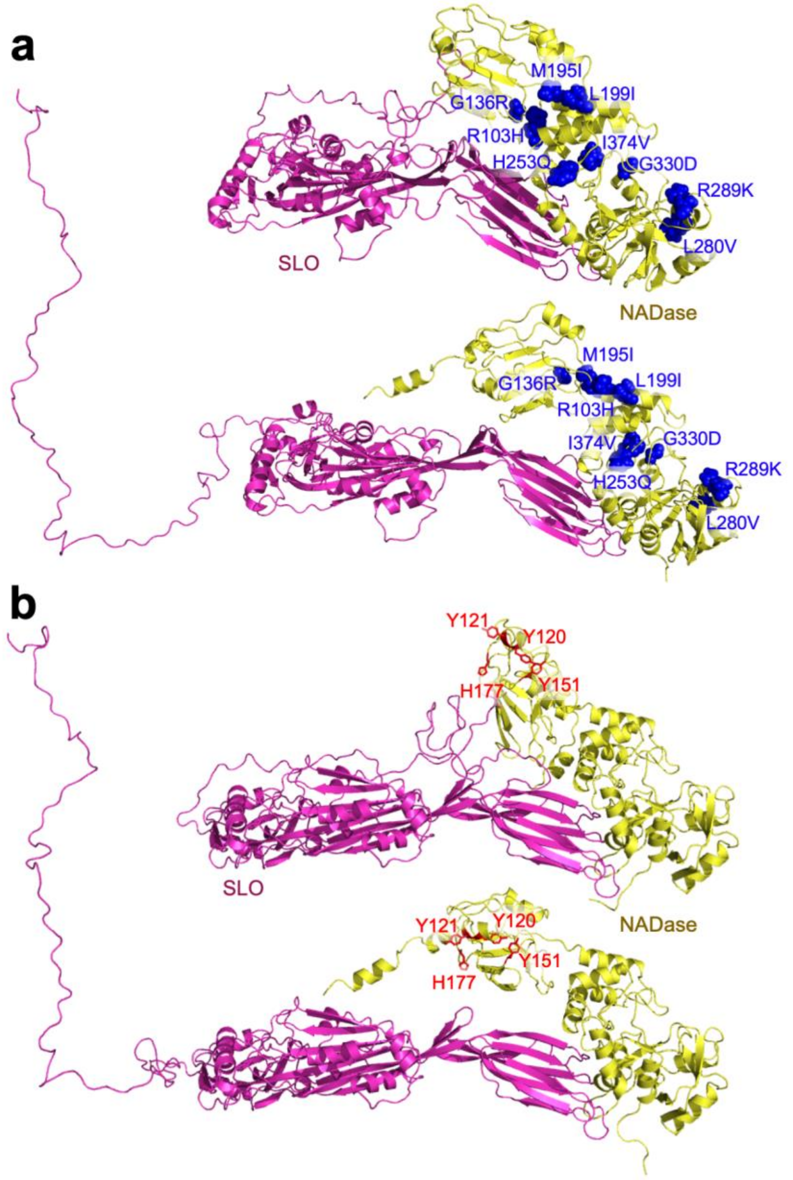

Supplementary Figure 8. Location of polymorphic residues and putative carbohydrate-binding residues of NADase in the NADase/SLO structural model. **a** Polymorphic residues of NADase[2] are highlighted by blue sphere. **b** Putative carbohydrate-binding residues[3], Tyr120, Tyr121, Tyr151, and His177, shown in red sticks are solvent-exposed in both the compact state (upper panel) and the extended state (lower panel).

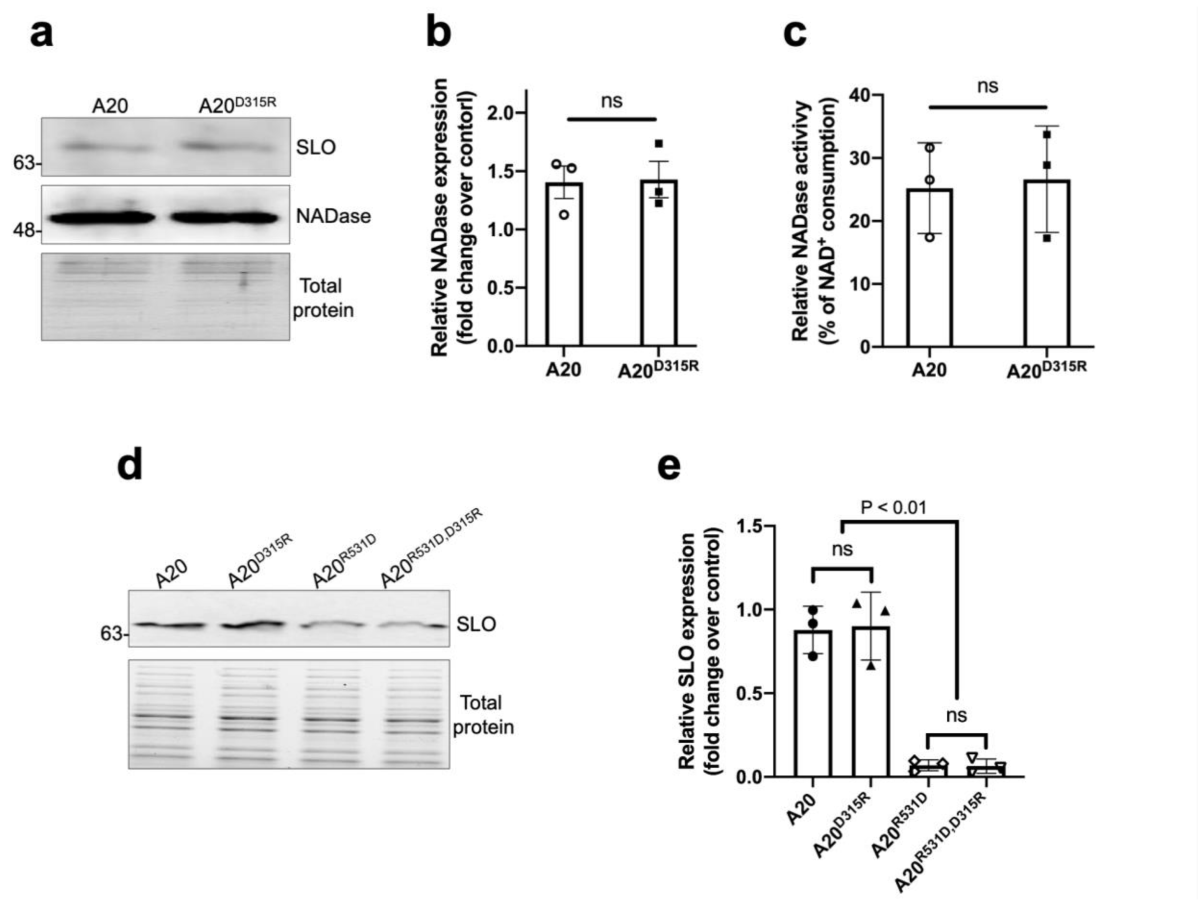

Supplementary Figure 9. Secretion levels of NADase and SLO in the A20 mutants. **a** Immunoblot of NADase and SLO in the culture supernatants of A20 and A20^{D315R}. Total protein represents the proteins of bacterial lysates in the corresponding cultures. **b** Densitometry of the immunoblots from **a**. NADase secreted from A20 and A20^{D315R} was quantified by band intensity presented as the ratio of NADase over total protein. **c** NADase activities in the culture supernatants of A20 and A20^{D315R}. **d** Immunoblot of SLO in the culture supernatants of A20, A20^{D315R}, A20^{R531D}, and A20^{R531D,D315R}. **e** Densitometry of the immunoblots from **d**. SLO secreted from A20, A20^{D315R}, A20^{R531D} and A20^{R531D,D315R} was quantified by band intensity presented as the ratio of SLO over total protein. Data are representative of three independent experiments as mean ± standard deviation, calculated by two-tailed unpaired t-test.

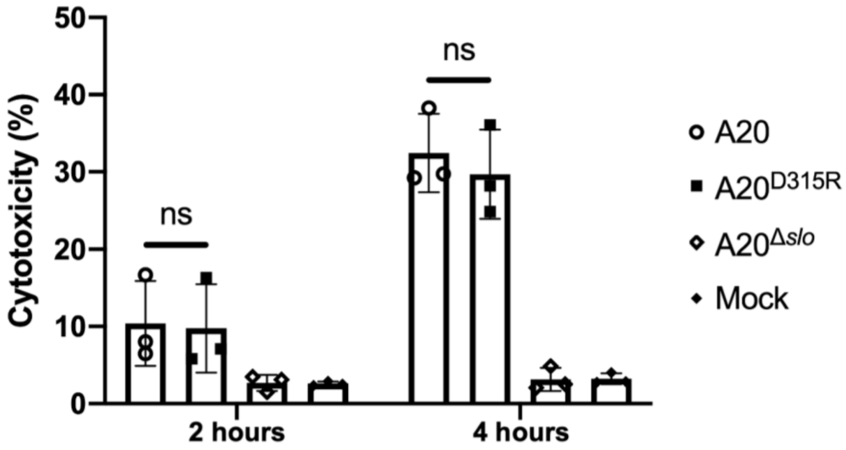

Supplementary Figure 10. Cytotoxicities of A20, A20^{D315R}, or A20$^{\Delta slo}$ on U937 cells. The cytotoxicities were measured at 2-hours and 4-hours post-infection. Cytotoxicities are presented as a percentage of lactate dehydrogenase released by uninfected cells lysed with 1% Triton X-100. Data from three independent experiments were analyzed by two-tailed unpaired t-test and shown as mean ± standard deviation.

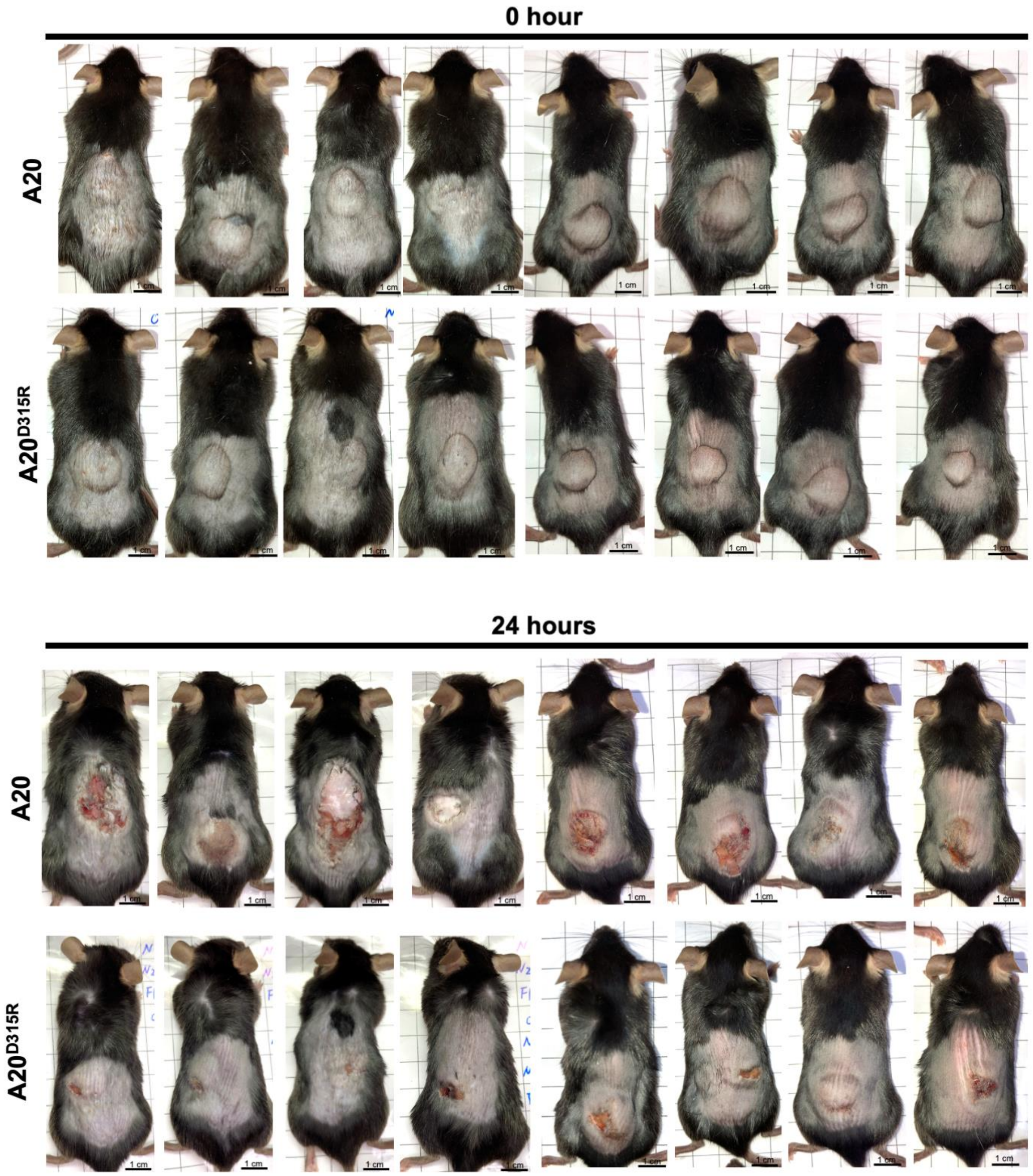

Supplementary Figure 11. GAS-infected mice. Images of GAS-infected air pouches (0 hours) and the necrotic skin lesions (24 hours). Scale bars: 1 cm.

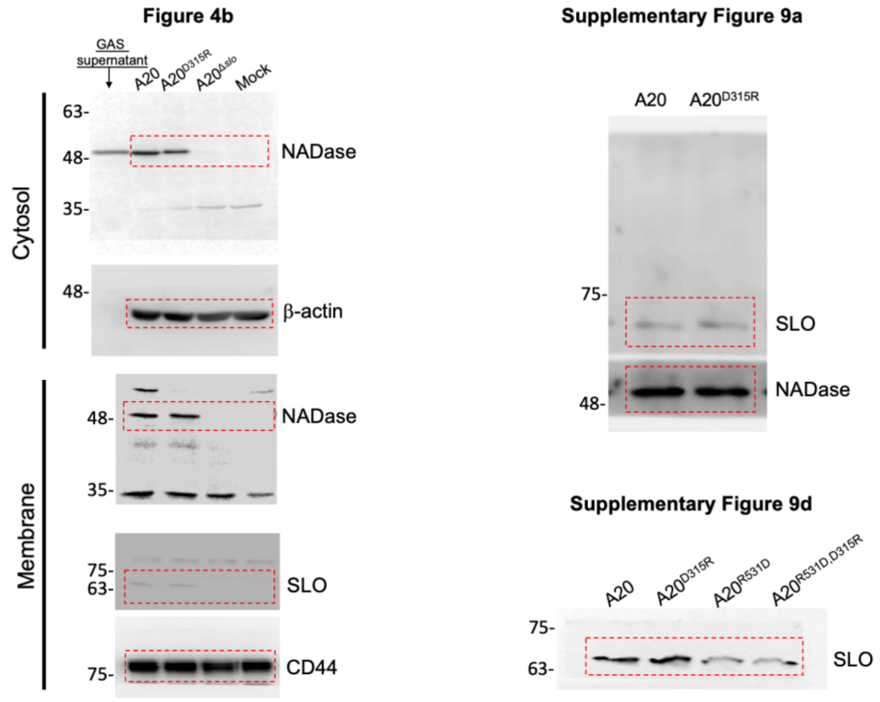

Supplementary Figure 12. Uncropped immunoblot images.

Supplementary References

1. Velarde, J. J., O'Seaghdha, M., Baddal, B., Bastiat-Sempe, B. & Wessels, M. R. Binding of NAD^+-Glycohydrolase to Streptolysin O Stabilizes Both Toxins and Promotes Virulence of Group A *Streptococcus*. *mBio* **8**, e01382-17, doi:10.1128/mBio.01382-17 (2017).
2. Chandrasekaran, S., Ghosh, J., Port, G. C., Koh, E. I. & Caparon, M. G. Analysis of polymorphic residues reveals distinct enzymatic and cytotoxic activities of the *Streptococcus pyogenes* NAD^+ glycohydrolase. *J Biol Chem* **288**, 20064-20075, doi:10.1074/jbc.M113.481556 (2013).
3. Velarde, J. J. *et al.* Structure of the *Streptococcus pyogenes* NAD^+ Glycohydrolase Translocation Domain and Its Essential Role in Toxin Binding to Oropharyngeal Keratinocytes. *J Bacteriol* **204**, e0036621, doi:10.1128/JB.00366-21 (2022).
