# Supplementary material for: Structural basis underlying the synergism of NADase and SLO during group A Streptococcus infection
Source: Commun Biol. 2023 Jan 31;6:124. doi: 10.1038/s42003-023-04502-0 (PMC9887584; doi:10.1038/s42003-023-04502-0)
Supplement: Supplementary file 3 — Description of Additional Supplementary Files [file 42003_2023_4502_MOESM3_ESM.pdf]

Description of Additional Supplementary Files

File name: Supplementary Data
Description: The source data behind the figures in the paper.
