# Supplementary material for: Structural basis underlying the synergism of NADase and SLO during group A Streptococcus infection
Source: Commun Biol. 2023 Jan 31;6:124. doi: 10.1038/s42003-023-04502-0 (PMC9887584; doi:10.1038/s42003-023-04502-0)
Supplement: Supplementary file 5 — Reporting Summary [file 42003_2023_4502_MOESM5_ESM.pdf]

nature portfolio

Corresponding author(s): Shuying Wang

Last updated by author(s): Dec 2, 2022

Reporting Summary

Nature Portfolio wishes to improve the reproducibility of the work that we publish. This form provides structure for consistency and transparency in reporting. For further information on Nature Portfolio policies, see our Editorial Policies and the Editorial Policy Checklist.

Statistics

For all statistical analyses, confirm that the following items are present in the figure legend, table legend, main text, or Methods section.

n/a	Confirmed	
☐	☒	The exact sample size (*n*) for each experimental group/condition, given as a discrete number and unit of measurement
☐	☒	A statement on whether measurements were taken from distinct samples or whether the same sample was measured repeatedly
☐	☒	The statistical test(s) used AND whether they are one- or two-sided<br>*Only common tests should be described solely by name; describe more complex techniques in the Methods section.*
☒	☐	A description of all covariates tested
☒	☐	A description of any assumptions or corrections, such as tests of normality and adjustment for multiple comparisons
☐	☒	A full description of the statistical parameters including central tendency (e.g. means) or other basic estimates (e.g. regression coefficient) AND variation (e.g. standard deviation) or associated estimates of uncertainty (e.g. confidence intervals)
☐	☒	For null hypothesis testing, the test statistic (e.g. *F*, *t*, *r*) with confidence intervals, effect sizes, degrees of freedom and *P* value noted<br>*Give P values as exact values whenever suitable.*
☒	☐	For Bayesian analysis, information on the choice of priors and Markov chain Monte Carlo settings
☒	☐	For hierarchical and complex designs, identification of the appropriate level for tests and full reporting of outcomes
☒	☐	Estimates of effect sizes (e.g. Cohen's *d*, Pearson's *r*), indicating how they were calculated

Our web collection on statistics for biologists contains articles on many of the points above.

Software and code

Policy information about availability of computer code

Data collection	Crystallographic datasets were collected on beamline TLS13B1, TLS15A1, and TPS05A at the National Synchrotron Radiation Research Center in Hsinchu, Taiwan. SAXS datasets were collected on beamline 12.3.1, Advanced Light Source, Lawrence Berkeley National Laboratory, Berkeley, CA, USA. SANS datasets were collected on the QUOKKA instrument at the Australian Nuclear Science and Technology Organization and were reduced using Igor Pro (v8.0) using NIST macros (v8.04).
Data analysis	Crystallographic data analyses were carried out using HKL2000, PHENIX (v1.18.2), Coot (v0.8.9.3), PISA (v1.52), and Pymol (v2.3.2). SAXS data were analyzed by SCÅTTER (v4.0), GNOM (v4.6), MODELLER, BILBOMD, FoXS, MultiFoXS (version main.3977adc), GASBOR (v2.3), DAMAVER, and CHIMERA (v1.13.1). SANS data analyses were performed using Primus (v3.4), GNOM (v4.6), CRYSON (v2.7), and MONSA (v1.45) from the ATSAS package. SEC-MALS data were analyzed using ASTRA 6 software (v6.1.7.17). Statistical analyses were performed using Prism 8 (v8.2.1). The image analyses of immunoblot and skin lesions were conducted using ImageJ (v1.53).

For manuscripts utilizing custom algorithms or software that are central to the research but not yet described in published literature, software must be made available to editors and reviewers. We strongly encourage code deposition in a community repository (e.g. GitHub). See the Nature Portfolio guidelines for submitting code & software for further information.

Data

Policy information about availability of data

All manuscripts must include a data availability statement. This statement should provide the following information, where applicable:

- Accession codes, unique identifiers, or web links for publicly available datasets
- A description of any restrictions on data availability
- For clinical datasets or third party data, please ensure that the statement adheres to our policy

Atomic coordinates and structure factors have been deposited in the Protein Data Bank (PDB code 7WVH). SEC-SAXS and SANS data were deposited in the SASBDB (codes SASDM47 and SASDM57). All data supporting the findings of the current study are provided in the paper and Supplementary Information. All additional information will be made available upon request to the authors.

Human research participants

Policy information about studies involving human research participants and Sex and Gender in Research.

Reporting on sex and gender	N/A
Population characteristics	N/A
Recruitment	N/A
Ethics oversight	N/A

Note that full information on the approval of the study protocol must also be provided in the manuscript.

Field-specific reporting

Please select the one below that is the best fit for your research. If you are not sure, read the appropriate sections before making your selection.

☒ Life sciences ☐ Behavioural & social sciences ☐ Ecological, evolutionary & environmental sciences

For a reference copy of the document with all sections, see nature.com/documents/nr-reporting-summary-flat.pdf

Life sciences study design

All studies must disclose on these points even when the disclosure is negative.

Sample size	The sample size is stated in the respective legends.
Data exclusions	No data were excluded.
Replication	All data presented in the manuscript are reproducible. The exact replicate number is stated in the legends.
Randomization	Not applicable.
Blinding	Not applicable.

Reporting for specific materials, systems and methods

We require information from authors about some types of materials, experimental systems and methods used in many studies. Here, indicate whether each material, system or method listed is relevant to your study. If you are not sure if a list item applies to your research, read the appropriate section before selecting a response.

Materials & experimental systems

n/a	Involved in the study	
☐	☒	Antibodies
☐	☒	Eukaryotic cell lines
☒	☐	Palaeontology and archaeology
☐	☒	Animals and other organisms
☒	☐	Clinical data
☒	☐	Dual use research of concern

Methods

n/a	Involved in the study	
☒	☐	ChIP-seq
☒	☐	Flow cytometry
☒	☐	MRI-based neuroimaging

Antibodies

Antibodies used

Anti-NADase (GTX64140, GeneTex), anti-Streptolysin O (ab188539, Abcam), anti-actin (MAB1501, Sigma-Aldrich), anti-beta-actin (A5441, Sigma-Aldrich), anti-CD44 (Cat. #3570, Cell Signaling Technology), anti-IL-1beta (AF-401, Novus Biologicals), anti-rabbit IgG (AB_2313567, Jackson ImmunoResearch), and anti-mouse IgG (ARG65350, Arigo Biolaboratories).

Validation

All antibodies used have been validated by the supplier for use in western blotting.

Eukaryotic cell lines

Policy information about cell lines and Sex and Gender in Research

Cell line source(s)

A549 (ATCC, CCL-185) and U-937 (ATCC, CRL-1593.2) were purchased from the cell bank of American Type Culture Collection.

Authentication

Cell lines were authenticated by supplier.

Mycoplasma contamination

Not tested.

Commonly misidentified lines
(See ICLAC register)

None of the cell lines used in this study is listed in the database of commonly misidentified cell lines maintained by iCLAC.

Animals and other research organisms

Policy information about studies involving animals; ARRIVE guidelines recommended for reporting animal research, and Sex and Gender in Research

Laboratory animals

Mice, C57BL/6, 8-10 weeks.

Wild animals

This study did not involve wild animals.

Reporting on sex

Female mice.

Field-collected samples

This study did not involve samples collected from the field.

Ethics oversight

All mice were maintained and handled according to the guidelines of the Institutional Animal Care and Use Committee (IACUC) of National Cheng Kung University, Tainan, Taiwan.

Note that full information on the approval of the study protocol must also be provided in the manuscript.
